# Apocrine Fibroadenoma of the Perianal Region Associated with Perianal Fistula

**DOI:** 10.4021/jocmr365w

**Published:** 2010-10-11

**Authors:** Baki Ekci, Ozcan Gokce, Ferda Ozkan

**Affiliations:** a Department of Surgery, Faculty of Medicine, Yeditepe University, Turkey; b Department of Pathology, Faculty of Medicine, Yeditepe University, Turkey

## Abstract

A case of a 45 year-old woman who presented with a perianal fistula was reported. Histologically, the excised lesion showed features of apocrine fibroadenoma. In addition, the lesion had both glandular and stromal growth patterns and active chronic inflammation in the background. Malignant or benign counterparts of these types of lesions are widely described in literature. However fistula does not usually accompany and is very rare. Therefore, it is crucial for the pathologists and clinicians to be aware of such lesions. The relationship between apocrine fibroadenoma and perianal region was also discussed.

**Keywords:**

Apocrin fibroadenoma; Perianal region; Fistula

## Introduction

Fibroadenomas are the most common benign tumors of the female breast, but adenomas in the anogenital region are uncommon [1, 2]. Ectopic breast tissue may occur anywhere along the primitive embryonic milk lines extending from the axilla to the groin [1-3]. Furthermore, it may also develop in male breast and extramammarian sites, including vulva and anogenital regions or other unusual locations. Female breast fibroadenomas are biphasic tumors consisting of both epithelial and stromal components [2]. However, the accompaniment of apocrine fibroadenoma and perianal fistula is rare. The aim of this case report was to present and discuss an unusual case of apocrine fibroadenoma of the perianal region associated with perianal fistula.

## Case Report

**Figure 1.**
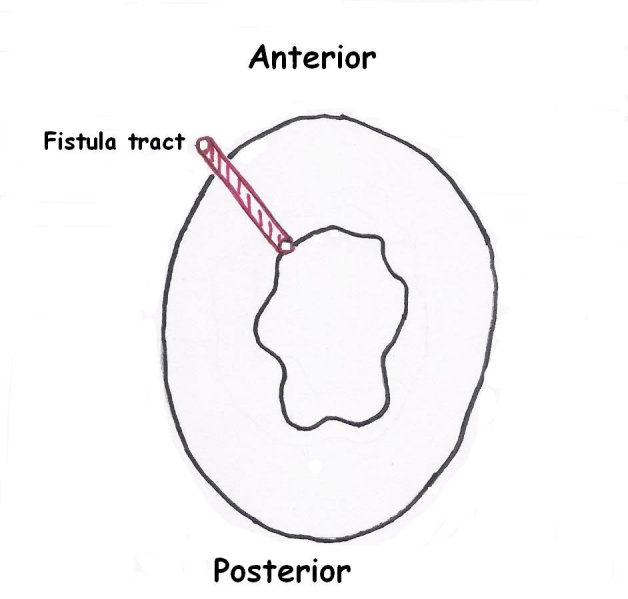
Localization of the perianal fistula tract.

**Figure 2.**
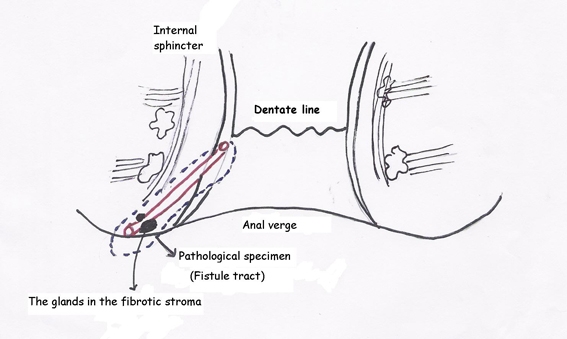
Perianal fistula tract in relation with anal canal.

A 45 year-old woman presented to the hospital with a chronic perianal fistula. During physical examination, a previous hemorroidectomy incision scar and a perianal fistula orifice at the end of this scar was observed both located at 11 o’clock of the anal verge (Fig. 1). The fistula was not in relationship with anal sphincter, and internal orifice of the fistula was located 2 cm distally in the anal canal (Fig. 2). Her past surgical history included hemorroidectomy one year ago. The patient’s family history was not significant. Fistulectomy was performed and the patient was discharged after the day of operation. The lesion was examined microscopically, and immunostaining was also performed. The pathological examination of the patient’s specimen revealed that the tissue sections exhibited a morphologic pattern similar to the mammary fibroadenoma.

### Histopathologic findings

**Figure 3.**
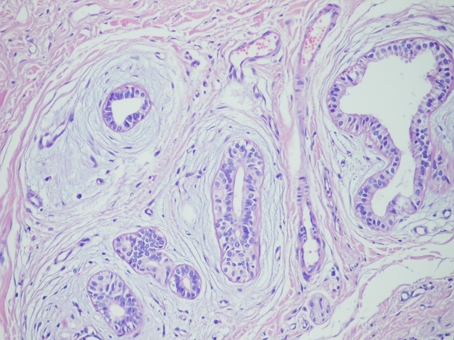
The glands arranged in a lobular pattern in the fibrotic stroma × 100 H & E.

**Figure 4.**
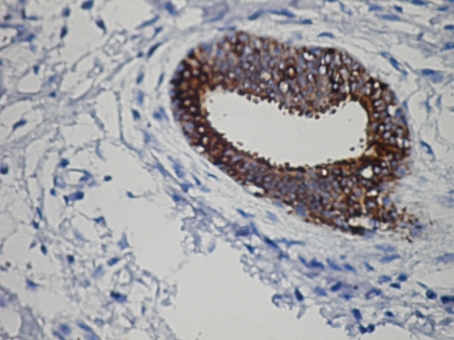
PR receptors in the lesion × 400 antibody against PR.

Grossly, 4 × 2 × 2 cm measuring specimen was composed of a skin piece sized 3 × 1 cm and a firm white fibrous subcutaneous tissue. The orifice of the fistula was observed on the skin and the fistula measured 2 cm in length at the subcutaneous tissue. The cut surface demonstrated bulging, firm, focally fibrotic tissue with cystic spaces. Microscopic tissue sections revealed an architectural pattern similar to the mammary fibroadenoma (Fig. 3). The glands were lined by a double layer of epithelial cells, an outer layer of relatively flat or cuboidal cells, and an inner layer of columnar cells with eosinophilic cytoplasm and focal prominent cytoplasmic snouts protruding into the lumen. Occasionally, glands were arranged in lobuli, resembling those of mammary glands. No nuclear pleomorphism was detected. The stroma appeared to be predominantly fibrotic. In some areas, especially surrounding the glandular ducts, the stroma was relatively loose. The epithelial cells expressed progesterone receptors, but did not express estrogen receptors and were negative for gross cystic disease fluid protein-15 (Fig. 4).

## Discussion

Fibroadenoma is one of the most common mammarian neoplasms. However, extramammarian fibroadenomas are quite rare and may occur in the anogenital region. Fibroadenoma is assumed to be the result of unopposed estrogenic stimulus on the susceptible tissue. Thus, anogenital fibroadenoma is the most common lesion emerging during the reproductive years, mostly less than 30 years of age [2]. The ectopic breast tissue may respond to hormones, and develop benign and malignant pathologic conditions such as fibrocystic disease, fibroadenomas, intraductal papillomas, and mucinous, ductal, and lobular carcinomas [3]. Etiological factors are not well-documented in the literature.

In 1991, van der Putte described the anogenital mammary-like sweat glands [4]. Anogenital mammary-like gland (MLG) arises from ectopic mammarian glands derived from the rudiments of the so-called embryonic milk line [1-3]. MLGs can occur on the atypical localizations such as vulva, perianal region, eyelid, nasal area, prostate gland and gallbladder [1-5]. These atypical localizations cannot support embryonic milk line theory, since these glands are considered to be the normal constituents of the anogenital region, and supernumerary mammary glands are not derived from the rudiments of the embriyonic regions [4]. Ectopic breast tissue may occur anywhere along the primitive embryonic milk lines, which extend from the axilla to the groin. They have many features in common with normal breast tissue, even in the expression of estrogen receptors and progesterone receptors and may develop benign or malign lesions [3]. These glands appear to be eccrine glands that are transformed fully or partially into apocrine glands and have histologic, biochemical, and ultrastructural features similar to that of apocrine glands [4]. In the present case, the lesion was positive for progesterone receptors, but negative for estrogen. There was also lobule formation. Located in the vulva, perineum and perianal areas, these glands have mixed ecrine, apocrine, and mammary gland histology [2, 4].

Fibroadenomas in the perianal region are usually recognized as indurations or tumors, and fistula is rare. The pathogenesis of this lesion is attributed to hyperplasia of stromal myofibroblasts in response to hormonal stimuli and the presence of myoepithelial cells testifies strongly toward its benignity [6]. Fibroadenomas arising in anogenital regions are histologically identical to their breast counterparts and can easily be differentiated by their unique morphology. Moreover, they are similar to the fibroadenoma of the breast and are usually seen as mass lesions in the anogenital region [2]. Their characteristic histology links and explains a series of disorders of the anogenital region in women (including functional vulvar mammary glands after pregnancy, fibrocystic disease, fibroadenoma, hidradenoma papilliferum, and some cases of adenocarcinoma, including Paget's disease), and in men (including fibroadenoma or apocrine cystadenoma, and cases of Paget's disease) [7]. In literature, only a 62-year-old man with extramammary Pagets disease in the scrotum was reported [8]. To our knowledge, this estrogen hormone-dependent gland may be seen in men but, it cannot be symptomatic. If a mass is present, establishing a morphological diagnosis with either tissue biopsy or aspiration cytology is recommended. Fine needle aspiration cytology has been accepted helpful in diagnosing such lesions. Excision or incision biopsy may be necessary for definitive diagnosis, and simple excision may be curative [2, 3]. It is important for pathologists and physicians to be familiar with mammary-like glands because they can give rise to several lesions, including fibrocystic disease, Paget’s disease, ductal carcinoma in situ and invasive carcinoma [2, 3].

In conclusion, the first case of an anal fibroadenoma in the perianal fistula tract was reported. To our knowledge, anogenital mammary-like sweat glands, known as the origin of most mammary-like lesions, may be found in this anatomic location. Therefore, occasional malignant ones may develop without apparent alarming features.
